# In-Depth Characterization of Zika Virus Inhibitors Using Cell-Based Electrical Impedance

**DOI:** 10.1128/spectrum.00491-22

**Published:** 2022-07-11

**Authors:** Merel Oeyen, Eef Meyen, Jordi Doijen, Dominique Schols

**Affiliations:** a Katholieke Universiteit Leuven, Department of Microbiology, Immunology and Transplantation, Rega Institute for Medical Research, Laboratory of Virology and Chemotherapy, Leuven, Belgium; Instituto de Histología y Embriología de Mendoza (IHEM)

**Keywords:** Zika virus, cellular assay, impedance, cell-based biosensor, antivirals

## Abstract

In this study, we use electric cell-substrate impedance sensing (ECIS), an established cell-based electrical impedance (CEI) technology, to decipher the kinetic cytopathic effect (CPE) induced by Zika virus (ZIKV) in susceptible human A549 lung epithelial cells and to evaluate several classes of compounds with reported antiviral activity (two entry inhibitors and two replication inhibitors). To validate the assay, we compare the results with those obtained with more traditional *in vitro* methods based on cell viability and viral yield readouts. We demonstrate that CEI can detect viral infection in a sensitive manner and can be used to determine antiviral potency. Moreover, CEI has multiple benefits compared to conventional assays: the technique is less laborious and better at visualizing the dynamic antiviral activity profile of the compounds, while also it has the ability to determine interesting time points that can be selected as endpoints in assays without continuous readout. We describe several parameters to characterize the compounds’ cytotoxicity and their antiviral activity profile. In addition, the CEI patterns provide valuable additional information about the presumed mechanism of action of these compounds. Finally, as a proof of concept, we used CEI to evaluate the antiviral activity of a small series of compounds, for which we demonstrate that the sulfonated polymer PRO2000 inhibits ZIKV with a response profile representative for a viral entry inhibitor. Overall, we demonstrate for the first time that CEI is a powerful technology to evaluate and characterize compounds against ZIKV replication in a real-time, label-free, and noninvasive manner.

**IMPORTANCE** Zika virus can cause serious disease in humans. Unfortunately, no antiviral drugs are available to treat infection. Here, we use an impedance-based method to continuously monitor virus infection in—and damage to—human cells. We can determine the Zika viral dose with this technique and also evaluate whether antiviral compounds protect the cells from damage caused by virus replication. We also show that this technique can be used to further unravel the characteristics of these compounds, such as their toxicity to the cells, and that it might even give further insight in their mechanism of antiviral action. Finally, we also find a novel Zika virus inhibitor, PRO2000. Overall, in this study, we use the impedance technology to—for the first time—evaluate compounds with anti-Zika virus properties, and therefore it can add valuable information in the further search for antiviral drugs.

## INTRODUCTION

Recent outbreaks with (re)emerging viruses such as Zika virus (ZIKV), dengue virus (DENV), Ebola virus, and severe acute respiratory syndrome coronavirus 2 (SARS-CoV-2) illustrate the enormous threat viruses pose to our global health system. ZIKV and DENV, among other pathogenic viruses such as yellow fever virus and West Nile virus, are positive-sense single-stranded RNA viruses belonging to the flaviviruses (genus: Flavivirus). ZIKV has been circulating for many decades without causing major threats but transformed to a “Public Health Emergency of International Concern” in 2015, when it caused a severe epidemic in South America (1). Its association with fetal microcephaly, as well as with other neurologic complications in adults, makes it a serious threat (2–4). Novel outbreaks of ZIKV remain possible since its transmission vector, Aedes mosquitoes, is able to increasingly spread to new areas due to several factors such as global warming and urbanization (5). Despite many promising efforts, no antiviral drug or vaccine to treat or prevent ZIKV infection has made it to the market yet.

Traditional methods to identify potential antiviral compounds are often based on the evaluation of cytopathic effects (CPE) in host cells after viral infection. These include the plaque assay, 50% cell culture infectious dose (CCID_50_) determination, or the determination of cell viability using colorimetric detection with, for example, the 3-(4,5-dimethylthiazol-2-yl)-5-(3-carboxymethoxyphenyl)-2-(4-sulfophenyl)-2H-tetrazolium (MTS) assay (6–8). The potency of the compounds can also be determined by assessing viral replication with quantitative PCR or by detecting viral antigens using immunofluorescence (9, 10). Assays using recombinant viruses that express reporter proteins are also commonly used, but to this end, viruses have to be genetically modified (11, 12). Usually, these assays are performed as endpoints that are labor intensive and/or that require complex analysis. Label-free real-time technologies are therefore attractive alternatives in the search for potential antiviral compounds.

Cell-based electrical impedance (CEI) is a label-free real-time technology that measures changes in impedance (*Z*) of a cell layer grown on a surface with embedded (gold) electrodes. The cells are exposed to an electric field generated by continuous sweeping of noninvasive alternating current (AC) voltages over a range of frequencies. As cells act as insulating particles in this system, they will resist the flow of AC, resulting in a frequency-dependent resistance measurement, also called impedance (*Z*). Changes in cell morphology, growth, or adhesion due to altered cell viability, migration, growth, spreading, proliferation, or any other change can lead to the current being more or less impeded. CEI has gained popularity in recent years and is used to monitor dynamic responses of cells toward receptor ligands, drugs, and pathogens. In this manner, CEI has been successfully implemented in cytotoxicity studies, cancer cell behavior, signaling pathway elucidation, and endothelial barrier function (13, 14).

Since many viruses cause CPE in susceptible cells, CEI can also be used in antiviral research to complement conventional antiviral assays. As cells grow, they spread out over the instrument’s electrodes. The growth of cells on the electrodes, together with the formation of tight junctions, will impede the current flow and subsequently will increase the measured impedance. On the other hand, as cells start to deform or detach due to viral infection, tight junctions will be disrupted, and the electrode surface will be covered with fewer cells or more loosely attached cells. This will allow more current passage and in turn will decrease the measured impedance (15–17). As such, CEI has been used to replace traditional CCID_50_ or plaque assays in virus titer determination (18). CEI has been implemented previously in antiviral research with various human and animal viruses such as influenza A virus, several herpesviruses, chikungunya virus, and SARS-CoV-2 (19–24). It can measure virus-induced changes and the activity of antivirals or neutralizing antibodies in real time, in a label-free manner, and in a medium-throughput setting. Nevertheless, the technology has been underexploited in the search for Flavivirus inhibitors. Fang et al. used CEI to study CPE induced by West Nile virus and St. Louis encephalitis virus and to quantify the protecting role of neutralizing antibodies (17). Cheng et al. determined that DENV-induced CPE was observed earlier with CEI than with conventional microscopy (25). However, to our best knowledge, the consequences of ZIKV infection on cellular impedance and the evaluation of ZIKV inhibitors have never been studied using CEI.

In the present study, we use electric cell-substrate impedance sensing (ECIS), an established CEI technology originally developed by Giaever and Keese (26, 27), to monitor cell growth and antiviral activity of various compounds after ZIKV (or DENV) infection in real time. We use ECIS to evaluate several compounds with described anti-ZIKV activity: the entry inhibitors labyrinthopeptin A1 (Laby A1) and duramycin (28, 29), as well as the polymerase inhibitors NITD008 (30) and 7-deaza-2′-*C*-methyladenosine (7DMA) (31). We compare the obtained ECIS results with traditional antiviral assays based on cell viability or virus yield to validate CEI as an antiviral tool. Furthermore, we use ECIS to determine various parameters to characterize the *in vitro* activity profile of antiviral compounds in more detail. Hereby, we validate CEI as a powerful tool to monitor ZIKV infection and to decipher the antiviral activity of compounds.

## RESULTS

### Real-time impedance monitoring of cell growth and ZIKV infection.

**FIG 1:**
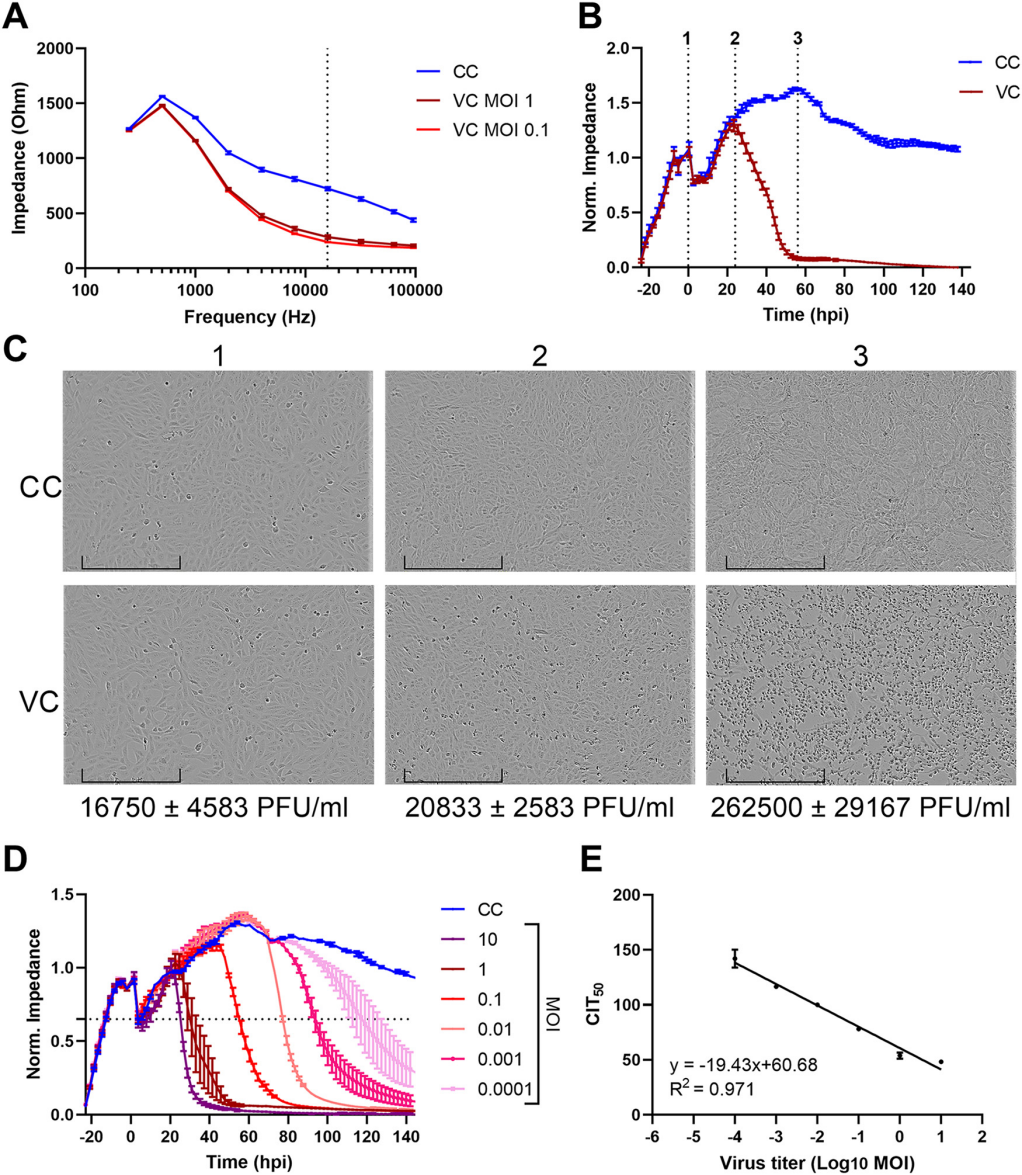
Comparison of impedance signal of uninfected and Zika virus (ZIKV)-infected A549 cells. (A) ECIS Bode diagram. The graph depicts the impedance signal of uninfected A549 cells (CC) and ZIKV-infected A549 cells (virus control [VC]; multiplicity of infection [MOI] 1 or MOI 0.1) at 100 h after seeding as measured in a frequency range of 250 to 96,000 Hz. The dotted line indicates the frequency at which the largest difference between CC and VC impedance was measured. This frequency (16,000 Hz) was used in all subsequent experiments. The means ± SD of four technical replicates are shown. (B) Kinetic normalized impedance pattern of uninfected control cells (CCs) compared to ZIKV-infected A549 cells (MOI 1; VC). ECIS data recorded at 16,000 Hz are shown. Dotted lines 1 to 3 indicate important observations at specific time points, which are further explained in the text. The graph represents means ± SD of four technical replicates. (C) Light microscopic pictures of uninfected (CC) and ZIKV-infected (VC) A549 cells at the different time points (time points 1 to 3) indicated in panel B. The pictures were taken with an IncuCyte S3. Bar, 400 μm. The viral titer at the specific time point was determined through plaque assay and is also indicated. The means ± range of three independent biological replicates performed in triplicate are shown. (D) Real-time monitoring of normalized impedance profile of uninfected control A549 cells (CC) and A549 cells infected with various MOI (0.0001 to 10) of ZIKV. The dotted line indicates the point at which the impedance decreases by 50%. The intersection of this line with the impedance curve depicts the time point at which impedance had decreased by 50% (CIT_50_) (× hours postinfection [hpi] + 24). The means ± range of two technical replicates are shown. (E) CIT_50_ values were regressed as a function of viral infectious dose (Log_10_ MOI). Regression equation and goodness of fit (*R*^2^) are shown. The means ± range of two technical replicates are shown.

ECIS instruments record impedance signals at a wide range of frequencies from 10 to 10^5^ Hz. First of all, we determined the optimal frequency to analyze our impedance patterns using the Bode diagram (25), which is a visualization of the impedance over different frequencies at a time point of choice. By looking at the Bode diagram at 100 h postseeding (or 76 hpi, when impedance of infected cells at multiplicity of infection [MOI] 1 or MOI 0.1 had dropped to baseline level), we can identify the frequency that leads to the largest observed impedance difference between uninfected and infected cells. When A549 cells, a commonly used cell line for ZIKV replication studies, were infected with ZIKV MR766 MOI 1 or MOI 0.1, the frequency with the largest difference was 16 000 Hz (Fig. 1A). In other words, the impedance is mostly affected by changes in cellular morphology due to ZIKV-induced cytopathology at this frequency. Therefore, 16,000 Hz was the chosen frequency for further testing and analysis.

In a first set of experiments, we monitored the impedance profile of uninfected and ZIKV-infected A549 cells. After seeding of the cells, impedance increases as a result of cell spread and adherence (Fig. 1B). At 24 h after seeding, the cells were infected with ZIKV MR766 MOI 1 (line 1 in Fig. 1B). The observed brief impedance dip shortly after treatment and infection is possibly the result of ECIS instrument and plate manipulation but needs further inquiry. Impedance of uninfected control cells further increases due to cell proliferation until a steady state is reached and during which the morphology or confluence of the cells no longer changes. In infected cells, impedance starts to decline at around 24 h postinfection (hpi) (line 2 in Fig. 1B) because the infection causes the cells to change morphologically (more cell rounding) and the impedance further declines until baseline level is reached around 60 hpi. At this time point, the cells are completely detached (line 3 in Fig. 1B). Of note, although the kinetic pattern might vary slightly between experiments due to variations in viral stock or cell states, the general observed pattern always follows the one depicted in Fig. 1B. The impedance changes after ZIKV infection correspond to the morphological changes observed microscopically at the indicated time points and also with increasing viral titers, as determined with plaque assay (Fig. 1C). This is the first time that the dynamic pattern of *in vitro* ZIKV infection has been monitored using impedance measurements.

Subsequently, A549 lung epithelial cells were infected with ZIKV MR766 at various MOI, and the impedance profile was constantly monitored for a week. As shown in Fig. 1D, the measured impedance changes as a function of virus input and time: the pattern shifts in time as cells have delayed CPE induction at lower viral MOI. The CEI assay detects ZIKV up to MOI 0.0001 during the evaluated time course. For each ZIKV dilution, the CIT_50_ was calculated. This value, specific for impedance measurements, refers to the time needed for the impedance signal to reduce by 50% (17). CIT_50_ was regressed as a linear function of the viral MOI (Log_10_ MOI). As indicated in Fig. 1E, for each order of magnitude increase in ZIKV MOI, the CIT_50_ is delayed by 19.4 h. Therefore, analysis of CEI patterns and CIT_50_ calculation of viral stock dilutions indicates the initial infectious dose of the ZIKV stock, as reported previously for other (flavi-)viruses (17, 18, 25, 30). This is an objective manner for performing virus titration, as no CPE scoring is needed. Furthermore, this kind of virus titration can be used to determine the most optimal time points to perform the readout of endpoint assays, such as cell viability evaluation, for a specific virus inoculum.

### Real-time impedance monitoring to evaluate compound cytotoxicity.

Four well-characterized inhibitors of ZIKV were included in this study: replication inhibitors 7DMA and NITD008 and entry inhibitors labyrinthopeptin A1 (Laby A1) and duramycin. It was demonstrated by our group and others that these inhibitors possess anti-ZIKV activity (28, 29, 31, 32). When assessing antivirals, it is important to first determine whether they induce cellular toxicity. The validation and usefulness of CEI measurements in cytotoxicity evaluation studies have been discussed before (33–36).

**FIG 2:**
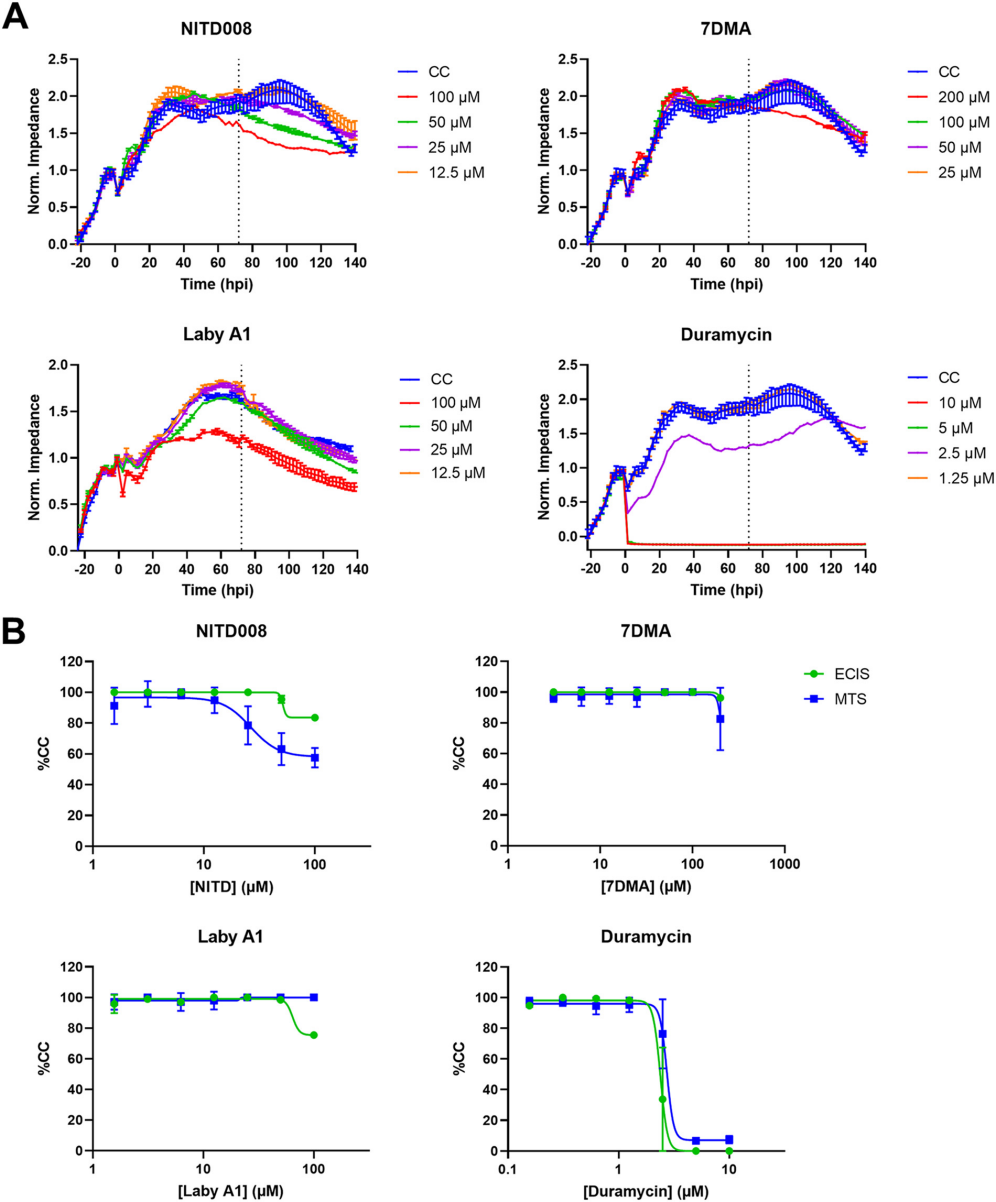
Cellular toxicity of various compounds when evaluated with ECIS. (A) Real-time evaluation of cellular toxicity using electric cell-substrate impedance sensing (ECIS). Confluent A549 cells were treated with various compound concentrations, and the impedance profile of the cells was continuously monitored using an ECIS Z array station. The experiment was performed in duplicate, three times independently. Graphs of a representative experiment are shown (means ± range of two technical replicates). (B) Comparison of cellular toxicity as evaluated with ECIS and 3-(4,5-dimethylthiazol-2-yl)-5-(3-carboxymethoxyphenyl)-2-(4-sulfophenyl)-2H-tetrazolium (MTS) viability assay, determined 72 h postincubation (indicated by the vertical lines in panel A). The percentage of compound treated cells compared to untreated cells was calculated. The results (means ± SD) of three independent experiments performed in duplicate are shown. The ECIS assay with NITD008 and 7DMA was performed two times in duplicate, so here the means ± range of two technical replicates are shown. 7 DMA, 7-deaza-2′-*C*-methyladenosine; Laby A1, labyrinthopeptin A1.

Adhered A549 cells were incubated with 2-fold serial dilutions of either NITD008, 7DMA, Laby A1, or duramycin, and the impedance was monitored over time (Fig. 2A). We compared cytotoxicity after incubating the cells with compounds for 72 h and compared it with the MTS viability assay that was also performed after 72 h of incubation with compound, as the latter is a commonly used method to determine compound cytotoxicity (Fig. 2B) (36). The impedance patterns of the highest concentrations of NITD008 and 7DMA deviate somewhat from cell control conditions, which is also reflected by MTS analysis. For Laby A1, ECIS seems to be slightly more sensitive than MTS analysis. This suggests that Laby A1 affects the cell morphology or adherence to some extent, without inducing cytotoxic changes. The highest concentration of duramycin (10 μM) leads to complete disruption of the cell monolayer almost immediately after adding the compound to the cells. This is also reflected in the MTS analysis. Interestingly, at 2.5 μM, the cell monolayer seems to recover slowly from duramycin treatment. This dynamic response to treatment is not observed when performing endpoint experiments but could in theory strongly affect the antiviral readout (because viral replication is compromised when cells are unhealthy). At 1.25 μM duramycin, cytotoxicity is no longer observed. The CC_50_, a useful parameter to determine cytotoxicity, can be determined by calculating the normalized area under the curve (AUC_n_). For NITD008 (CC_50_ > 100 μM), 7DMA (CC_50_ > 200 μM), and Laby A1 (CC_50_ > 100 μM), CC_50_ cannot be determined as the compounds do not induce substantial cellular toxicity at the highest concentration tested. The CC_50_ of duramycin is 3.6 ± 0.2 μM.

Hence, the ECIS assay supports our results obtained with the MTS viability assay while also providing a better understanding of the compounds’ cellular effects. The latter can help explain why particular compounds might appear antiviral while instead they are altering the cell’s state, which could indirectly lower virus replication as well. The opposite is also true: when compounds do not alter the monolayer in the absence of virus (at least at the concentrations used in the antiviral experiments), the cellular changes observed in the presence of virus relative to the virus control are solely due to the impact the compound has on the virus. The inhibition of the virus effect at these concentrations is therefore due to antiviral activity of the compounds and not due to cytotoxicity. The real-time data and the holistic nature of the readout obtained with CEI are other considerable advantages, since signs of aberrant cell morphology can be early indications of cytotoxicity or undesired side effects. Also, as some compounds become toxic only after a certain incubation period, this can be more easily overlooked by endpoint assays.

### Validation of real-time impedance monitoring as antiviral tool.

**FIG 3:**
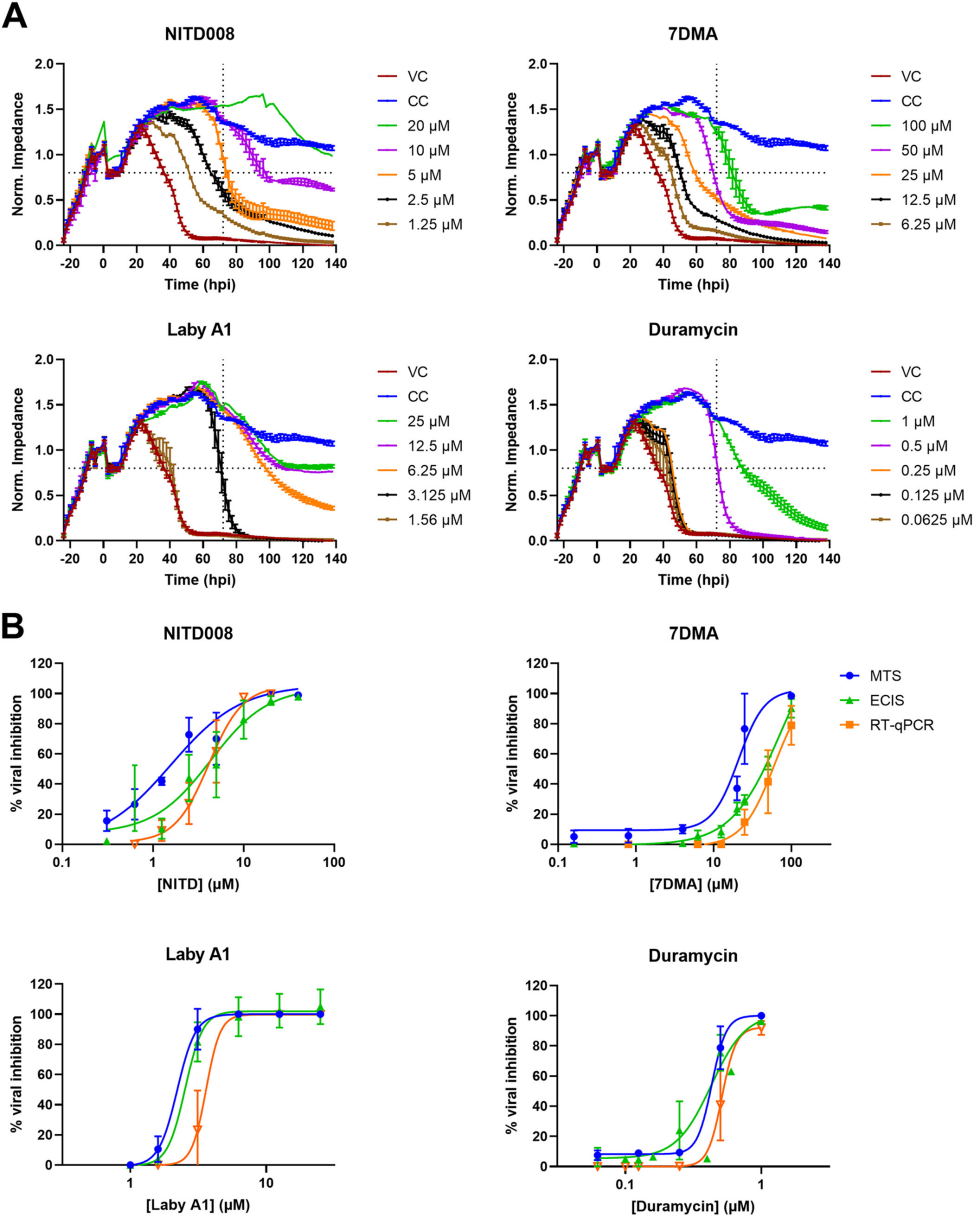
ECIS profiles of anti-ZIKV activity of various compounds and comparison with conventional methods. Seeded A549 lung epithelial cells were incubated with 2-fold dilutions of compound, followed by infection with ZIKV MR766 at MOI 1. Impedance was monitored for a week. (A) Graphical representation of the normalized impedance pattern in function of time is shown. Dotted vertical lines indicate the 72-hpi time point at which IC_50_ was calculated. The dotted horizontal lines indicate the CIT_50_, the time point (× hpi + 24) at which impedance has dropped by half (compared to the maximal CC value). The experiment was performed three or four times, and the graphical results of one representative experiment performed in duplicate are shown (means ± range). (B) Dose-response curves of inhibition percentages of different compounds across various assays. Inhibition of viral replication was determined 72 hpi either by determining cell viability using MTS (blue), by quantifying viral replication using RT-qPCR (orange), or by measuring impedance using ECIS (green). Percentages of viral inhibition of each compound concentration was compared to CC (100%) and VC (0%). The results of three or four independent experiments performed in duplicate are shown (means ± SD of three or four biological replicates). 7DMA, 7-deaza-2′-*C*-methyladenosine; Laby A1, labyrinthopeptin A1.

We then used these inhibitors to evaluate the use of CEI as antiviral tool. After overnight incubation, A549 cells were pretreated with 2-fold serial compound dilutions and subsequently infected with ZIKV at MOI 1. Nontoxic serially diluted concentrations were used (as determined with MTS viability determination) (Fig. 2). Impedance was monitored continuously for 144 h after treatment. As shown in Fig. 3A, all compounds inhibit the virus-induced decrease in impedance, and this in a dose-dependent manner. Interestingly, we observed a time-dependent antiviral activity. The antiviral activity of the compounds decreases over time. This can be explained by the incomplete blocking of ZIKV replication at a certain compound concentration, which leads to delayed detection of viral replication. Our results demonstrate that the chosen time point of compound potency evaluation will affect this potency. Therefore, CEI is a useful tool to determine at which time points it makes the most sense to perform endpoint assays.

This dynamic antiviral activity of the test compounds is undetected with traditional endpoint assays, unless several assays with different endpoints are performed or multiple sample collections are implemented, which consumes more time and money. Although this time-dependent effect is not often evaluated in other studies, it has been observed previously (37).

To validate the use of CEI as an antiviral tool in ZIKV research, we compared the IC_50_ of ZIKV inhibition as evaluated with the ECIS assay with the IC_50_ obtained with more traditional antiviral assays; i.e., based on cell viability (MTS readout) and on viral replication (reverse transcriptase quantitative PCR [RT-qPCR] readout) at 72 hpi (dotted vertical lines in Fig. 3A and B; Table 1). There are some significant differences (*P < *0.05) between the IC_50_ values, but overall, our results confirm that these potencies are comparable, and their ranking is the same between the different methods. Therefore, we conclude that CEI can also be used as a tool to evaluate compounds as ZIKV inhibitors. Our calculated values closely resemble potencies of published work, although other cell lines or viral strains might have been used here (29, 31, 32).

**TABLE 1:** Comparison of the anti-ZIKV activity of a set of selected compounds as evaluated with ECIS, MTS, and RT-qPCR^*a*^

Compound	72-hpi ECIS	MTS		RT-qPCR		AUC_n_ ECIS	
	IC_50_ ± SD (μM)	IC_50_ ± SD (μM)	*P*	IC_50_ ± SD (μM)	*P*	IC_50_ ± SD (μM)	*P*
NITD008	3.0 ± 1.6	2.8 ± 1.8	0.88	4.1 ± 1.7	0.42	3.5 ± 1.6	0.60
7DMA	41.7 ± 13.4	14.6 ± 6.9	0.01^*b*^	60.4 ± 26.5	0.24	48.5 ± 11.2	0.34
Laby A1	2.4 ± 0.5	2.2 ± 0.1	0.33	3.3 ± 1.3	0.10	3.4 ± 0.8	0.01^*b*^
Duramycin	0.4 ± 0.1	0.5 ± 0.1	0.56	0.6 ± 0.2	0.05	0.6 ± 0.1	0.01^*b*^

*^a^*The table shows the potency of different compounds against Zika virus (ZIKV) MR766 with a multiplicity of infection (MOI) of 1 in A549 cells. The ECIS IC_50_ calculated at 72 hpi was compared with the other groups by using two-tailed unpaired *t* test. The mean IC_50_ values ± SD of three to five biological replicates are shown. hpi, hours postinfection; ECIS, electric cell-substrate impedance sensing; IC_50_, 50% inhibitory concentration; MTS, 3-(4,5-dimethylthiazol-2-yl)-5-(3-carboxymethoxyphenyl)-2-(4-sulfophenyl)-2H-tetrazolium; RT-qPCR, reverse transcriptase quantitative PCR; AUC_n_, normalized area under the curve; 7DMA, 7-deaza-2′-*C*-methyladenosine; Laby A1, labyrinthopeptin A1.

^*b*^*P* value < 0.05.

Because CEI has the advantage of monitoring the cells in real time, one can also make use of a parameter that includes this continuity of the data to determine the compounds’ potencies. Therefore, we calculated the AUC_n_ for every compound concentration and also accompanying dose-response curves (Fig. S1). The IC_50_ was calculated compared to control cells (CCs) and virus controls (VCs) (Table 1). Although there are some significant differences, these results are in line with those calculated at the endpoint that is traditionally used in our antiviral ZIKV assays (72 hpi). Therefore, AUC is an accurate parameter for potency determination of antivirals.

### Benefits of real-time impedance monitoring for a more in-depth characterization of antiviral compounds.

**FIG 4:**
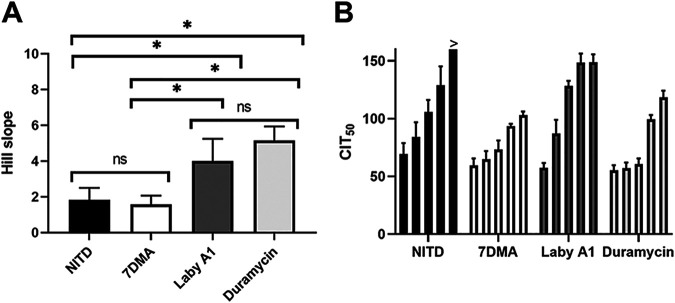
Comparison of replication and entry inhibitor parameters. A549 cells were treated with various compound dilutions and infected with ZIKV MR766 MOI 1. Impedance was monitored for a week. (A) AUC_n_ was calculated, and dose-response curves were obtained using the nonlinear regression four-parameter fitting tool (shown in Fig. S1 in the supplemental material). Hill slopes were calculated and compared using a two-tailed unpaired *t* test. *P* values < 0.05 are indicated with asterisks. ns, not significant. The means ± SD of four or five independent experiments performed in duplicate are shown. (B) CIT_50_ was calculated for every concentration. The means ± SD of three or four independent experiments performed in duplicate are shown. Compound concentrations for each set of bars from left to right are as follows: NITD008: 1.25, 2.5, 5, 10, and 20 μM; 7DMA: 6.25, 12.5, 25, 50, and 100 μM; Laby A1, 1.56, 3.125, 6.25, 12.5, and 25 μM; duramycin: 0.0625, 0.125, 0.25, 0.5 μM, and 1 μM; PRO2000: 3.125, 6.25, 12.5, 25, and 50 μM. The > symbol indicates that no CIT_50_ could be calculated since this compound concentration was still inhibiting virus replication at the end of the experiment. 7DMA, 7-deaza-2′-*C*-methyladenosine; Laby A1, labyrinthopeptin A1.

We were also interested in the use of CEI to further characterize antiviral compounds. For example, is it possible to discriminate between entry or replication inhibitors based on their impedance pattern? When the Hill slopes of the AUC_n_ dose-response curves shown in Fig. S1 are compared, there is a significant difference between the replication inhibitors NITD008 and 7DMA and the entry inhibitors Laby A1 and duramycin (Fig. 4A). This observation is also reflected in the CIT_50_, another parameter that next to AUC_n_ takes into account of the dynamic properties of continuous impedance measurements. Active compounds prevent or delay viral replication and therefore prolong the time to get a decrease in impedance level, resulting in a larger CIT_50_. Indeed, as can be deduced from Fig. 3 and is also shown in Fig. 4B, the CIT_50_ increases with increasing compound concentrations, since higher compound concentrations can better delay viral replication. Fig. 4B suggests that the CIT_50_ of the replication inhibitors NITD008 and 7DMA increases gradually, while a more sudden jump in CIT_50_ is observed for the evaluated entry inhibitors Laby A1 and Duramycin. Indeed, when we plot CIT_50_ of the experiment depicted in Fig. 3A as a function of log (compound concentration), we observe that for the evaluated replication inhibitors, the graphs fit to a straight line, while the fitting of the evaluated entry inhibitors improves when a logistic sigmoidal regression is applied (Fig. S2). However, due to high CIT_50_ variability (possibly by changes in, for example, viral stock and cell passage), it is impossible to approve this observation as statistically significant. Although the observations should be interpreted with extreme caution, they are of particular interest since it suggests that CEI profiles can potentially classify inhibitors according to their presumed mechanism of action. Of course, it is also possible that these findings are consequences of the chemical and/or pharmacokinetic properties of the compounds, with NITD008 and 7DMA being small molecules, while Laby A1 and duramycin are peptides. Therefore, more compounds should be included to strengthen these observations. Hence, in future experiments, small molecule inhibitors that target viral entry and peptide inhibitors that target viral replication will be included. As shown below under “CEI assay reveals PRO2000 as ZIKV inhibitor,” we confirm the observations using another viral entry inhibitor, PRO2000.

**FIG 5:**
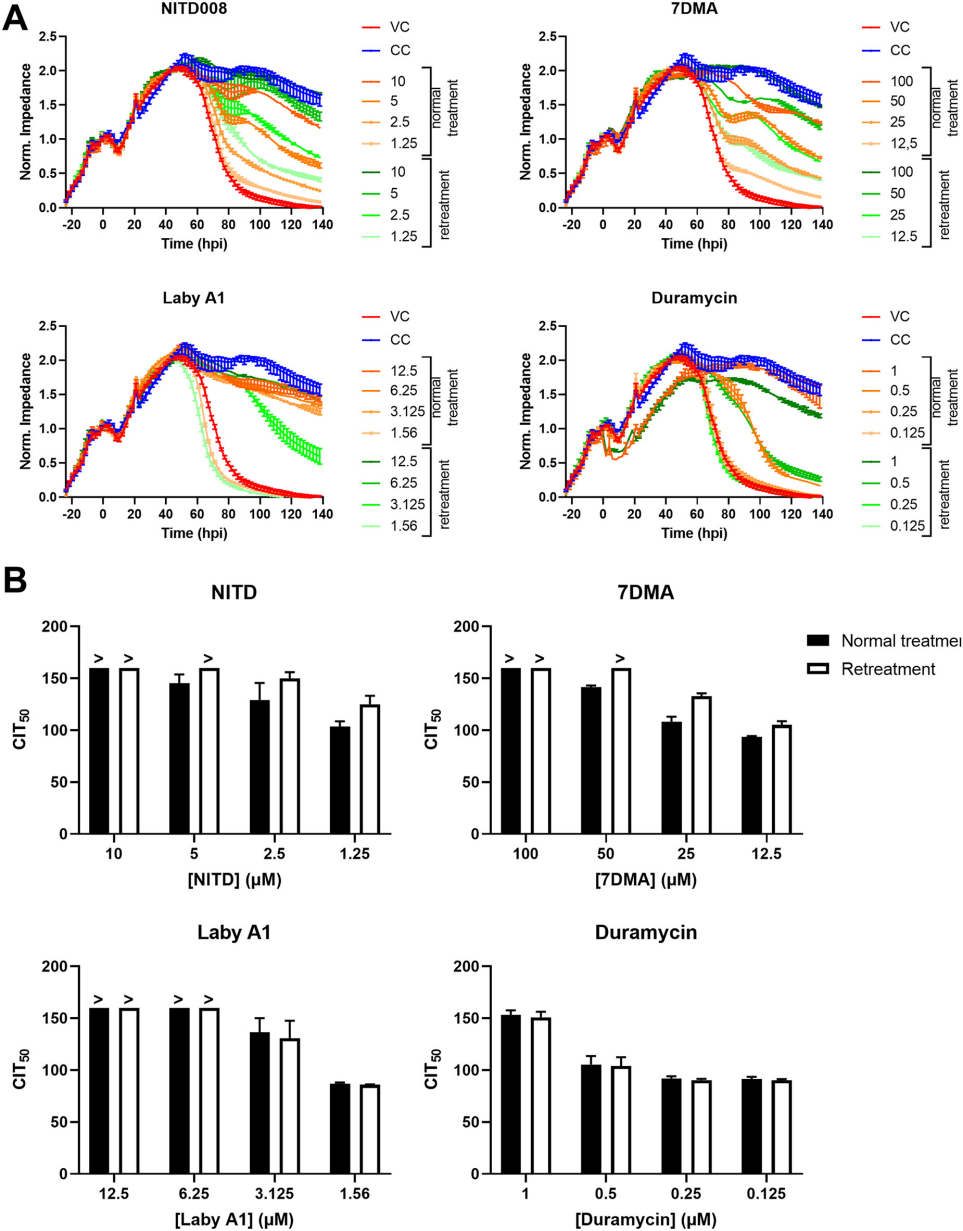
When cells are retreated with compound, this leads to a CIT_50_ shift for replication inhibitors but not for entry inhibitors. Adhered A549 cells were infected with ZIKV MR766 MOI 0.1. The samples were either treated once before infection (at 0 hpi; normal treatment) or twice, before infection and at 24 hpi (Retreatment). (A) Impedance profiles (means ± range of two technical replicates) of a representative experiment. (B) For each compound concentration, CIT_50_ was calculated. The > symbol indicates that no CIT_50_ could be calculated as the compound concentration still inhibited viral replication at the end of the experiment. The means ± SD is shown of three independent experiments performed in duplicate. 7DMA, 7-deaza-2′-*C*-methyladenosine; Laby A1, labyrinthopeptin A1.

In a next set of experiments, we wanted to further exploit the real-time measurements of CEI. We wondered if it is possible to categorize entry and replication inhibitors based on their CEI response to repetitive treatment. Therefore, we evaluated whether repetitive treatment extended the antiviral activity of the compound or, in other words, increased CIT_50_ values. The cells were treated for a first time before infection, and 24 h after infection, compound treatment was repeated (without intermediate washing steps). Impedance patterns and CIT_50_ values were compared with those of a classic infection with treatment solely prior to infection. As shown in Fig. 5A, the impedance pattern of NITD008 and 7DMA shifts for the various concentrations, as the green graphs (retreatment) slow down the virus-induced CPE compared to the orange graphs (normal treatment). This is also illustrated by the CIT_50_ values: CIT_50_ increases somewhat for the replication inhibitors NITD008 and 7DMA, while it remains unaltered for the entry inhibitors Laby A1 and duramycin (Fig. 5B). In other words, adding additional compound has less pronounced effects for Laby A1 and duramycin, while the activity of NITD008 and 7DMA is prolonged. The presence of a CIT_50_ shift for NITD008 and 7DMA is possibly due to their mechanism of action. Because Laby A1 and duramycin intervene in the viral life cycle at an early point, it might be of less use to retreat cells if the virus has already entered the cells and completed its life cycle. The ZIKV life cycle is completed 24 hpi, so the progeny has already started to replicate (38). Other explanations related to chemical properties of the compounds are also plausible. For example, another equally likely possibility is that NITD008 and 7DMA are metabolically inactivated and degrade more quickly. Therefore, to further investigate and confirm this observation in more detail, more compounds with distinct chemical properties will be evaluated in the future. This kind of assay might be useful in preclinical studies to give insight in treatment regimens.

**FIG 6:**
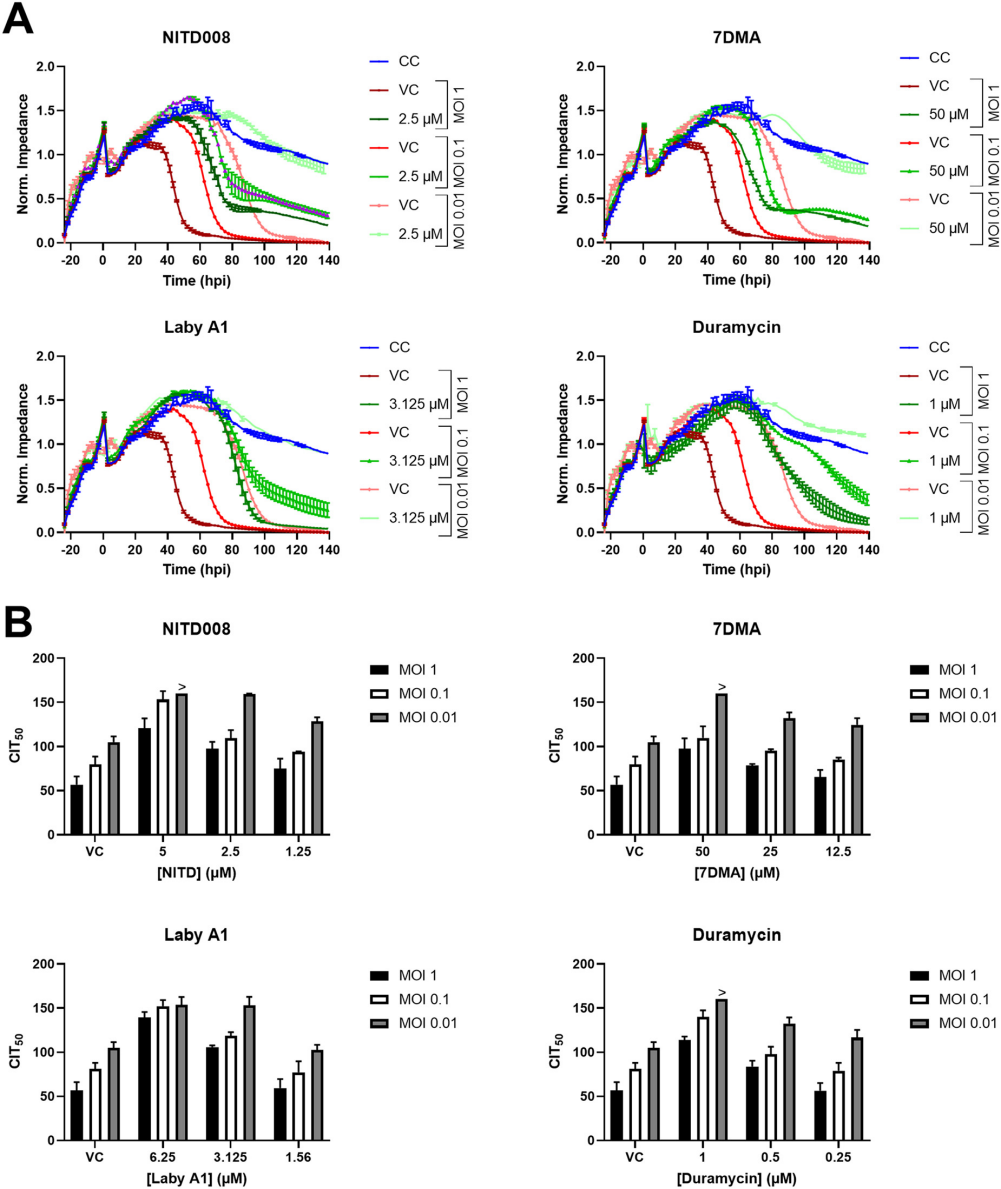
Correlation of MOI and CIT_50_. Confluent A549 cells were treated with compound and infected with ZIKV MR766 at various MOIs, and the impedance was monitored. (A) ECIS patterns of a representative experiment with two technical replicates (means ± range). For clarity reasons, only VC and one compound concentration at different MOI are shown. (B) Mean CIT_50_ ± SD of three independent experiments. The > symbol indicates that no CIT_50_ could be calculated since the impedance of the compound concentration had not decreased by 50% at the end of the experiment. 7DMA, 7-deaza-2′-*C*-methyladenosine; Laby A1, labyrinthopeptin A1.

Finally, we were also curious whether the compound’s CIT_50_ depends on the initial viral dose. Therefore, A549 lung epithelial cells were incubated with compound concentrations and infected with ZIKV MR766 at various MOI. As depicted in Fig. 6B, infection at a lower MOI leads to a higher CIT_50_ for the same compound concentration. This is expected, since the more viral particles are present, the sooner all cells will be infected in the presence of partially blocking compound concentrations, and the faster CPE can be observed. However, this set of experiments mainly shows that CEI is useful to determine the compound’s dependency of infectious dose. As an example, Fig. 6A demonstrates that for a certain compound concentration, ZIKV-induced CPE is completely blocked at an MOI of 0.01 throughout the whole impedance monitoring, while virus-induced CPE is only delayed at higher MOI. This is important to keep in mind, since in many antiviral assays, the antiviral effect of compounds is only evaluated at a certain MOI (39–41).

### CEI assay reveals PRO2000 as ZIKV inhibitor.

**FIG 7:**
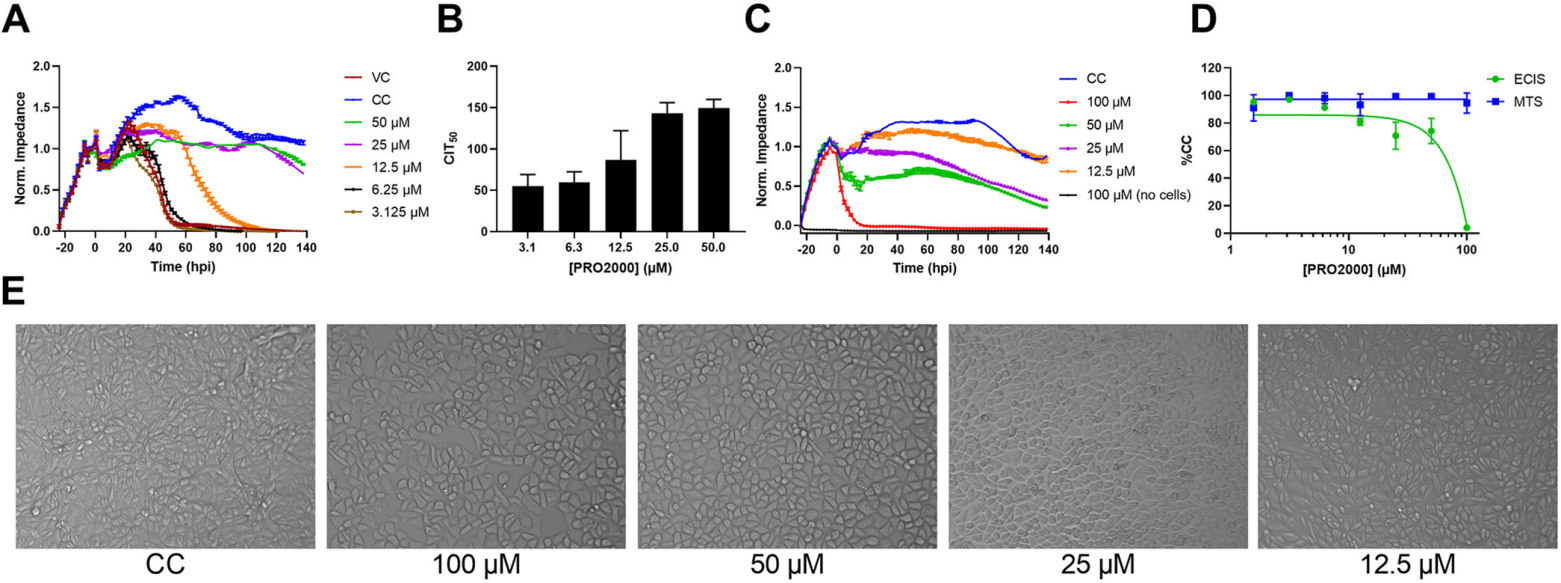
PRO2000 inhibits ZIKV-induced CPE. (A) Impedance profile of A549 cells treated with PRO2000 and infected with ZIKV MR766 (MOI 1). The results of one representative experiment performed in duplicate (means ± range) are shown. (B) CIT_50_ was calculated for every concentration. The means ± SD of three independent experiments performed in duplicate are shown. (C) Cytotoxicity impedance profile of PRO2000 on A549 cells. The results of one representative experiment performed in duplicate (means ± range) are shown. (D) Comparison of MTS and ECIS cytotoxicity evaluation. The means ± SD of three independent experiments performed in duplicate are shown. (E) Light microscopic images of untreated or PRO2000-treated A549 cells at 100, 50, 25, and 12.5 μM.

Because we have already validated CEI as a useful tool to identify and characterize potential ZIKV inhibitors, we used the CEI assay to evaluate additional compounds with published antiviral activity (Table S1). We demonstrate that the previously described HIV entry inhibitor PRO2000 (42) prevents ZIKV-induced cell death (Fig. 7A). The IC_50_ was 12.3 ± 4.3 μM according to AUC_n_ and 13.8 ± 6.2 μM at 72 hpi. We confirmed the antiviral activity of PRO2000 with MTS (IC_50_ = 14.8 ± 3.9 μM) and with RT-qPCR (IC_50_ = 5.1 ± 0.6 μM). As shown in Fig. 7B, the CIT_50_ values increase with increasing concentrations. The CIT_50_ dose-response graph fits a sigmoidal curve, and the Hill slope of the AUC dose-response curve is 5.4, which is in line with the other evaluated entry inhibitors. There is a surprising discrepancy when the cytotoxicity of PRO2000 is evaluated with ECIS compared to MTS (Fig. 7C and D). At concentrations up to 100 μM, PRO2000 is not cytotoxic according to MTS evaluation, while these concentrations have pronounced effects on the cell layer as measured with ECIS (CC_50_ based on AUC_n_ = 55.5 ± 3.0 μM). This suggests that PRO2000 affects the cell morphology or cellular adherence or has cytostatic properties without directly leading to cell death (as seen with the cytotoxic duramycin). In Fig. 7E, microscopic images of PRO2000-treated cells indeed show that cells look different after treatment with the highest compound concentrations. The cell number does not change significantly after PRO2000 treatment (data not shown). Fig. 7C also shows that the negatively charged polymeric PRO2000 does not interact with the ECIS sensor when no cells are present (black curve).

## DISCUSSION

In this study, we demonstrate the power of implementing cell-based electrical impedance measurements in the search for Flavivirus inhibitors. The CEI assay offers valuable information when monitoring morphological cell changes in response to ZIKV infection in real time, in a label-free manner, and in a noninvasive assay format. Impedance measurements can be used to quantify viral load, and the kinetic readout can help determine at which time points endpoint assays should be performed. We used CEI to—for the first time—determine the antiviral activity of a set of previously described ZIKV entry and replication inhibitors. The assay can be used to determine compound cytotoxicity, as well as the compound’s dynamic antiviral activity profile, and this with the same metric. Finally, we also aimed to categorize compounds based on impedance features that might be representative of their mechanism of action. Additional sets of compounds should be tested in the future to assess whether the assay can indeed be used to differentiate between entry blockers and later stage antivirals.

Phenotypic assays are routinely used in antiviral research by both academic groups and the pharma industry. In general, the reduction in CPE or plaque formation after compound treatment is evaluated, for example, microscopically or with a luminescent, fluorescent, or colorimetric readout. However, these endpoint assays are very often time consuming and possess other drawbacks; they require multiple handling steps, the onset of CPE or the kinetics of the infection cannot be detected, and it is crucial that the optimal endpoint is chosen in order to achieve a good assay quality. CEI, on the contrary, offers a simple noninvasive and label-free workflow, with real-time measurements and an objective/unbiased readout. Furthermore, the samples can be further processed after the impedance experiment is finished; since the method is noninvasive, qPCR can be performed on both cells and supernatant fractions, while the cells can also be lysed for Western blot (WB) analysis. Moreover, after the impedance run, staining can be performed, and the plates can be imaged. The usefulness of the CEI technology in the search for antivirals has been reported previously for other pathogenic viruses (19, 20, 22, 23). The main limitation of the ECIS assay (and other CEI assays) is related to the equipment needed: the ECIS device consists of a well station that is placed in a cell culture incubator, a control module, and a computer equipped with the analyzing software. Furthermore, specific microtiter plates embedded with gold electrode sensors are required, increasing the cost of the assay. The instrument’s throughput might also represent an issue. During the whole duration of the experiment, no other assays can be run. Of course, the experiment can also be run as an endpoint assay to increase throughput, by measuring the samples’ impedance only during the time window of interest. Hence, kinetic data are not provided. Because of the rather low throughput, CEI is not attractive in a compound screening setting. Including sufficient controls and replicates, up to 45 compound samples can be monitored simultaneously on a 96-well format. However, since CEI is becoming increasingly popular, the ECIS equipment’s throughput might by increased in the future. Agilent, another provider of CEI technology (xCELLigence RTCA), already provides 384-well formats. Despite the throughput, CEI has other valuable applications in a preclinical research. We suggest that it could be implemented either in an initial phase to facilitate high-throughput screening (e.g., by determining interesting time points) and in later stages to complement current methodologies during further selection and characterization of novel antivirals (i.e., during lead optimization and mechanism of action studies).

More recent models of the ECIS instrument allow the direct measurement of the two impedance components: resistance and capacitance. These are interesting parameters as they provide additional biological information about the different cellular behaviors at play, with resistance being more prone to changes in cell-cell contacts, while capacitance rather represents cell-substrate interactions and cell surface coverage (16). When both parameters are monitored, this could potentially reveal additional information on ZIKV infection biology, in addition to the currently described CPE measurement.

Impedance-based biosensors are also currently emerging to complement traditional diagnostic tools in virology such as enzyme-linked immunosorbent assay (ELISA) and RT-qPCR (43, 44). Here, impedance measurements also have some advantages over the more traditional assays, as they provide a sensitive, label-free, and real-time readout without requiring laborious sample preparation. For diagnostic purposes, a target-specific biorecognition element is often implemented in the sensor. Impedance-based biosensors have been developed for the specific detection of ZIKV or other flaviviruses, where virus-specific capture antibodies or probes have been immobilized to the electrodes of the sensor (45–47). However, the course of *in vitro* ZIKV infection and the evaluation of inhibitors have never been studied. Therefore, this study further demonstrates the capabilities of this technology for virus studies, while also adding new insights to Flavivirus antiviral research.

Using the CEI assay, we identified PRO2000 as a novel ZIKV inhibitor. The assay also indicated that PRO2000 has pronounced effects on the cell monolayer, albeit in a nontoxic way. Previous research has shown that PRO2000 potentially has multiple mechanisms of action against HIV, since it—in addition to interacting with HIV glycoprotein gp120—also interacts with the CD4 and CXCR4 cell membrane receptors, both HIV entry receptors (48). It has also been shown previously that PRO2000 inhibits cell-to-cell transmission of HTLV-1 virus (49). PRO2000 is a polyanionic polymer, indicating that the negative charges could possibly interact with positive charges of various cell surface proteins. These cellular interactions and multiple targets could explain the altered impedance response that we observed. Although our results show that PRO2000 has pronounced effects on the cells’ behavior *in vitro*, clinical trials with PRO2000 have demonstrated that it is safe for therapeutic use in humans (50).

Like previous studies that have used CEI for virus research (16, 19, 23), we further demonstrate how this label-free kinetic readout can complement more traditional methods and more specifically how it can help researchers to better evaluate their selected lead agents. Our work demonstrates that when different methods complement each other, new insights in the field of antiviral research can be gained.

## MATERIALS AND METHODS

### Cell lines, primary cells, and virus strains.

**(i) Cell lines.** Human lung carcinoma A549 cells and baby hamster kidney (BHK-21) cells were obtained from ATCC (Manassas, VA, USA) and grown in minimum essential medium (MEM; Thermo Fisher Scientific [TFS], Waltham, MA, USA) supplemented with 10% fetal bovine serum (FBS), 2 mM l-glutamine (TFS), and 0.075% sodium-bicarbonate (TFS). The mosquito cell line C6/36 (isolated from Aedes albopictus) was obtained from ATCC and cultured in Leibovitz’s L-15 medium (TFS) supplemented with 10% FBS, 0.01 M HEPES (TFS), and penicillin/streptomycin (TFS). Cell cultures were maintained at 37°C in a humidified environment with 5% CO_2_, except for C6/36 cells, which were cultured at 28°C in the absence of CO_2_. The cells were passaged every 3 to 4 days.

**(ii) Viruses.** ZIKV prototype strain MR766 (isolated from sentinel Rhesus monkey, Uganda, 1947) was obtained from ATCC. ZIKV was propagated in C6/36 cell cultures from which supernatant containing the virus was harvested 5 to 9 days after infection and stored at −80°C. Viral titers were determined using plaque assays in BHK-21 cells, as described below. All viruses were obtained and used as approved according to the rules of a Belgian institutional review board (Departement Leefmilieu, Natuur en Energie, protocol SBB 219 2011/0011n) and the Biosafety Committee at the Katholieke Universiteit Leuven.

### Antiviral test compounds.

Labyrinthopeptin A1 (2,073.7 Da) was isolated and purified as described earlier (51). Duramycin and NITD008 were purchased from Sigma-Aldrich (St. Louis, MO, USA). 7-Deaza-2′-C-methyl-d-adenosine (7DMA) was purchased from Carbosynth (Berkshire, UK). PRO2000 (molecular weight: ~5,000 g/mol) was kindly provided by A.T. Profy (formerly at Indevus Pharmaceuticals Inc., Lexington, MA, USA).

### CPE reduction and cytotoxicity assays.

Test compounds were screened for their antiviral activity using a colorimetric antiviral assay that was originally described by Pauwels et al. (52). We adapted the protocol as described in more detail by Van Hout et al. (53). A549 cells were seeded in cell culture medium in a 96-well plate at 15 × 10^3^ cells/well and allowed to adhere and grow overnight. The next day, serial dilutions of compound were prepared in cell culture medium without FBS (final FBS concentration was 4%), followed by 30 min of incubation. Then, ZIKV was added at a multiplicity of infection (MOI) of 1. Negative controls (cell controls [CCs]) were mock infected with culture medium and DMSO. After 3 incubation days, supernatant was collected and stored at −80°C for further analysis with RT-qPCR. CPE was determined microscopically, and the cell viability was determined using the spectrophotometric MTS/phenazine ethosulfate (PES) viability staining assay (Cell-Titer 96 Aqueous one solution Proliferation assay kit; Promega, Madison, WI, USA). Absorbance was measured at 498 nm using the Versamax microplate reader and analyzed using SoftMax Pro software (Molecular Devices, Sunnyvale, CA, USA). To determine whether the compound itself induced cellular toxicity, the assays were also performed without the addition of virus. The 50% inhibitory concentration (IC_50_), which is defined as the compound concentration that is required to inhibit virus-induced CPE by 50%, and the 50% cytotoxic concentration (CC_50_), which is defined as the compound concentration required to reduce the cell viability by 50%, were determined. Each experiment was performed in duplicate.

### ECIS assay.

The electric cell-substrate impedance sensing (ECIS) Z array station (Applied Biophysics, Troy, NY, USA) was used to measure changes in electrical resistance across the cell monolayer during incubation with compounds and viral infection. ECIS plates with interdigitated electrodes (96W20idf PET) were washed prior to use with culture medium for 4 h at 37°C. Next, medium was replaced by an A549 cell suspension (15 × 10^3^ cells/well). The growth of the cells was monitored overnight in the ECIS Z array station in multifrequency (MFT) mode at 37°C in 5% CO_2_. In MFT mode, the electrodes are probed with a weak and noninvasive alternating current (AC) signal at 11 frequencies between 10 and 10^5^ Hz to measure the frequency-dependent impedance (*Z*). Changes in *Z* that are representative for cell adhesion and proliferation were measured for 24 h. The next day, compound dilutions were added in culture medium without FBS (final FBS concentration was 4%), followed by infection with ZIKV MR766 at various MOI. Negative controls (CC) were mock infected with culture medium and DMSO. The measurement was continued in MFT mode for the subsequent 5 days. Every ECIS experiment lasted 160 h in total: 24 h with cells only and the remaining time with compound and virus. Measurements were taken every 8 min. The data were further processed using Microsoft Excel (Microsoft, Redmond, WA, USA). During every experimental run, each sample was measured through time and in duplicate, and the mean *Z* ± SD at every time point was calculated. Impedance is reported at 16,000 Hz, because at this frequency, the difference in impedance between VC and CC is most pronounced 3 days postinfection (Fig. 1A).

### Viral plaque assay.

BHK-21 cells were seeded in 12-well plates in growth medium (4 × 10^5^ cells/well). The next day, the cells were incubated in triplicate with either 10-fold viral stock dilutions or with cell supernatant dilutions collected at different hours postinfection. Assay medium was included as a negative control. After 1 h, medium was replaced with a microcrystalline cellulose overlay (Avicel RC 581, IMCD Benelux, Mechelen, Belgium) and incubated for 4 days. The overlay was removed, after which the cells were fixed in 70% ethanol and stained with crystal violet solution (Merck, Darmstadt, Germany). The plaques were counted, and the infectious viral titer was determined according to the following formula: number of plaques × dilution factor × (1/inoculation volume).

### RNA isolation and quantitative RT-PCR.

Virus lysis, RNA isolation, and RT-qPCR were performed using the CellsDirect one-step RT-qPCR kit (Thermo Fisher Scientific) according to the manufacturer’s instructions. During RT-qPCR, the ZIKV E protein encoding region (nucleotides 1193 to 1269) was amplified using the primers 5′-CCGCTGCCCAACACAAG-3′ (forward) and 5′-CCACTAACGTTCTTTTGCAGACAT-3′ (reverse), together with a Double-Quenched Probe 5′-6-FAM/AGCCTACCT/ZEN/TGACAAGCAATCAGACACTCAA/3′ IBFQ (54, 55). Primers and probes were obtained from Integrated DNA Technologies (IDT, Leuven, Belgium). The viral copy numbers were quantified based on a standard curve produced using serial 10-fold dilutions from viral DNA templates with known concentrations.

### Real-time IncuCyte assay.

A549 cells were seeded (15 × 10^3^ cells/well), and after overnight incubation, they were infected with ZIKV MR766 at various MOIs or left untreated. The cells were incubated and imaged in real time at 37°C for 4 days in an IncuCyte S3 (Essen BioScience Inc., Ann Arbor, MI, USA). Images were taken every 8 h, with five fields imaged per well under ×4 magnification.

### Data analysis.

The impedance data were normalized (*Z*′) according to the following formula:

(1) $$Z\prime =\;\frac{\left(Z_{\text{x}}\;-\;Z_{\text{end}}\right)}{\left(Z_{\text{0 hpi}}\;-\;Z_{\text{end}}\right)}$$

The impedance at the time just prior to infection (*Z*_0 hpi_) was set to a value of 1, and the impedance at the end of the experiment (160 h; *Z*_end_) was set to a value of 0, while the impedance of the intervening time points (*Z*_x_) were scaled relative to *Z*_0 hpi_ and *Z*_end_. GraphPad Prism (version 9.2.0, GraphPad Software Inc., San Diego, CA, USA) was used to fit the normalized data into a curve. When quantifying the compounds’ antiviral activity against ZIKV, various parameters were calculated to allow evaluation and comparison of the compound potencies: CIT_50_, AUC_n_, and IC_50_. CIT_50_ was calculated as the time point at which impedance had decreased 50%, compared to the maximum impedance of the cell control. The area under the normalized curve (AUC_n_) was calculated over the whole duration of the experiment (160 h). This parameter represents the growth and viability of the cells after seeding, treatment, and/or infection. The percentage inhibition of a compound at a particular concentration in both ECIS and CPE reduction assays was obtained by subtracting the negative-control (cell control [CC]) response followed by normalizing to the positive-control (virus control [VC]) response. Dose-response curves were obtained with GraphPad Prism using the nonlinear regression four-parameter fitting tool. IC_50_ values were used to represent the potency of the compounds and calculated according to the following formula:

(2) $${\text{IC}}_{50}={\text{exp}}^{\left[\ln\left(\text{C}1\right)-\left(\text{ln}\left(\frac{\text{C1}}{\text{C2}}\right)\;.\;\frac{\left({\text{\%inhib}}_{\text{C}1}-50\right)}{\left({\text{\%inhib}}_{\text{C1}}-{\text{\%inhib}}_{\text{C}2}\right)}\right)\right]}$$

Here, C1 is the compound concentration resulting in more than 50% inhibition, C2 is the compound concentration resulting in less than 50% inhibition, with their respective percentage of inhibition (%inhib_C1/C2_). To compare IC_50_ values or Hill slopes, two-tailed unpaired *t* tests were used. *P* values < 0.05 were considered significant. The quality of the ECIS assay was determined using the *Z*′ factor, based on AUC_n_ values between 0 and 160 h after seeding (19, 56). This was calculated using following formula:

(3) $$Z\prime =1-3*\frac{\left({\text{σ}}_{\text{vc}}\;+{\text{σ}}_{\text{cc}}\right)}{\left|{\text{μ}}_{\text{vc}}\;-{\text{μ}}_{\text{cc}}\right|}$$

where σ_vc_ and σ_cc_ correspond to AUC_n_ standard deviations of VC and CC, respectively; and μ_vc_ and μ_cc_ correspond to AUC_n_ means of VC and CC, respectively. All performed assays in our study had *Z*′ factors above 0.5, which is considered an excellent screening assay value (56) (data not shown).
