# Supplementary material for: In-Depth Characterization of Zika Virus Inhibitors Using Cell-Based Electrical Impedance
Source: Microbiol Spectr. 2022 Jul 11;10(4):e00491-22. doi: 10.1128/spectrum.00491-22 (PMC9431523; doi:10.1128/spectrum.00491-22)
Supplement: Supplemental file 1 — Supplemental material. Download spectrum.00491-22-s0001.pdf, PDF file, 0.4 MB [file spectrum.00491-22-s0001.pdf]

Supplementary Table

Supplementary Table 1 Anti-ZIKV IC_{50} values and CC_{50} values of various evaluated compounds in A549 cells using the ECIS assay, as calculated by AUC_n.

Compound	AUC_n IC_{50}	AUC_n CC_{50}	Reference[3]
Chloroquine[1]	37/>50	>50	[1]
Curcumin	>100	58.0	[2]
Heparin	>50 µg/ml	>50 µg/ml	[3]
Hippeastrum hybrid lectin	>50	>50	[4]
Nanchangmycin[2]	>1	>1	[5]
Obatoclax[1]	1/>1	>1	[6]
PRO2000	12.3 ± 4.3	55.5 ± 3.0	[7]
Remdesivir	>100	>100	[8]
Ribavirin[2]	>200	>400	[9]
Sofosbuvir	>50	>50	[10]
Suramin	>100	>100	[3]

Confluent A549 cells were treated compound prior to infection with ZIKV MR766 MOI 1 and impedance was monitored for a week. Compound treatment in the absence of virus was monitored in parallel to define cytotoxicity. Results from 1-2 independent experiments performed in duplicate are shown. [1]Although the antiviral impedance pattern of Chloroquine and Obatoclax suggest that these compounds are inhibiting ZIKV replication, their antiviral impedance curves closely resembled their cytotoxicity curves. Therefore, the observed antiviral activity might be due to the inability of the virus to replicate in damaged cells.

[2]For Nanchangmycin and Ribavirin, a dose-dependent impedance shift was observed, but no 50% inhibition of ZIKV replication was reached according to AUC_n calculation. Furthermore, Nanchangmycin exerted cytotoxic effects at the highest concentrations tested.

[3]These compounds were selected because they have described antiviral activity against ZIKV or other enveloped viruses, which is indicated in the reference.

Supplementary Figures

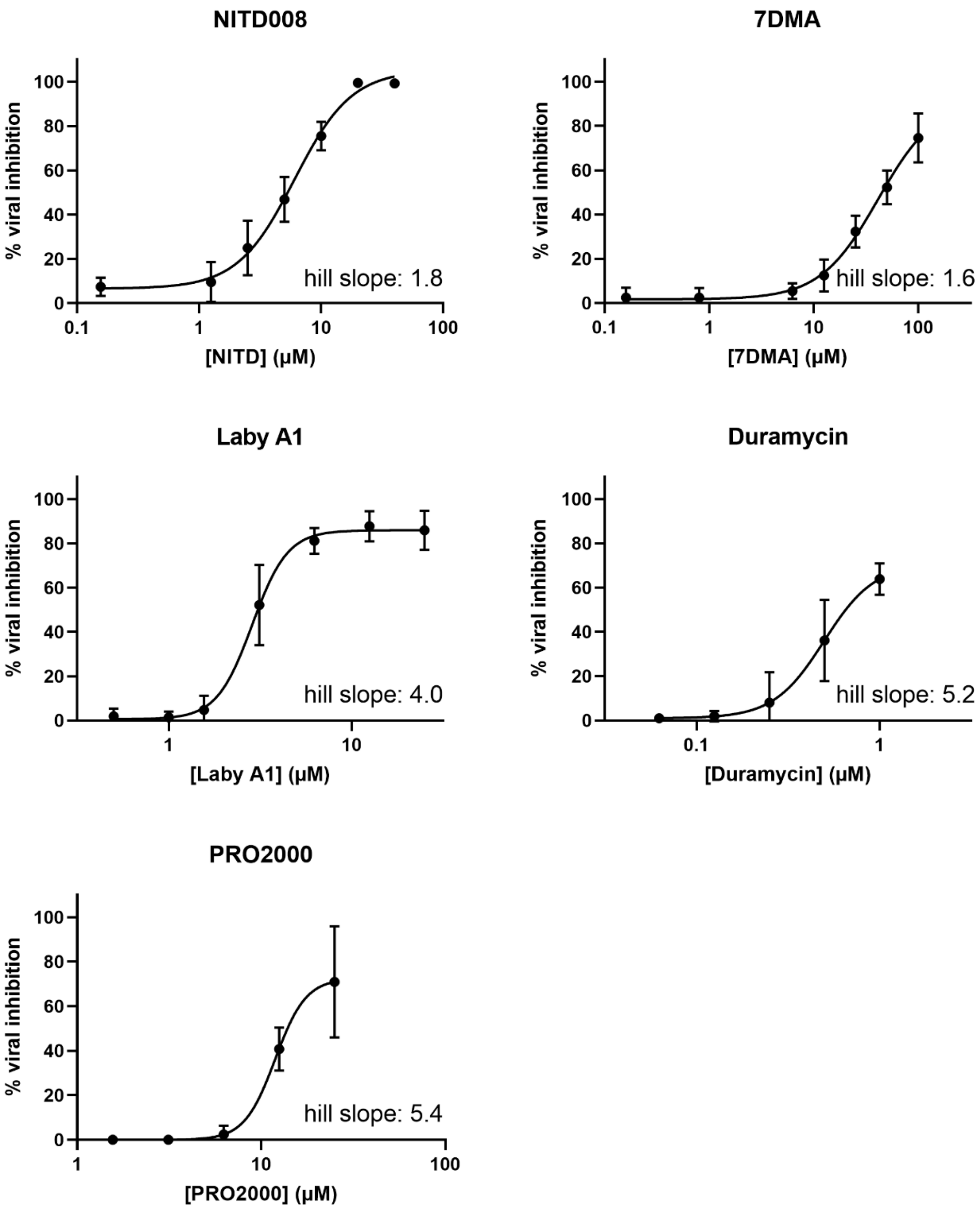

Supplementary Figure 1 Dose-response curves of AUC_n. A549 cells were treated with various compound dilutions and infected with ZIKV MR766 MOI 1. Impedance was monitored for a week. AUC_n was calculated and dose-response curves were obtained using the nonlinear regression four-parameter fitting tool. Mean ± SD of 4-5 independent experiments performed in duplicate is shown.

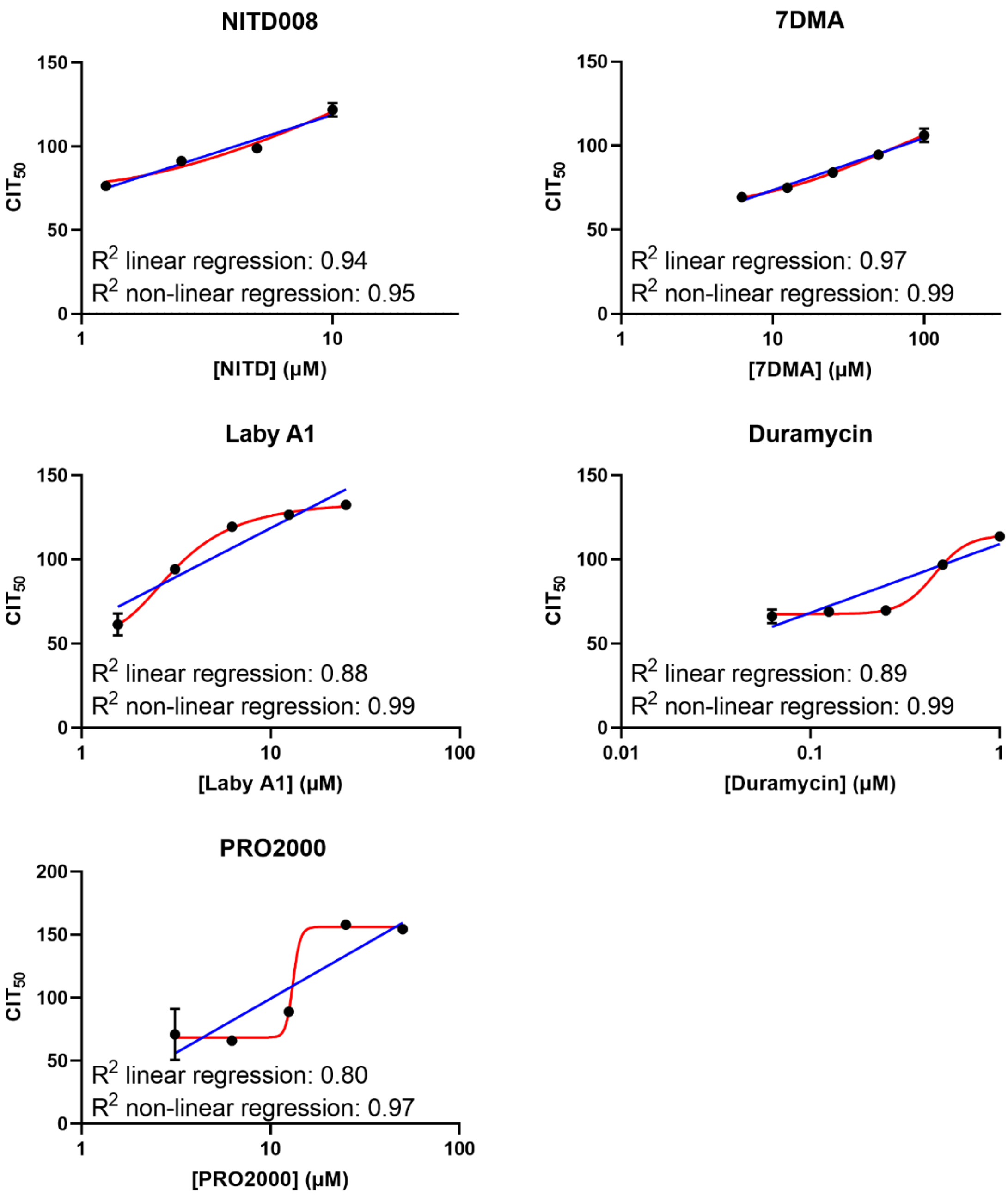

Supplementary Figure 2 Could ECIS patterns be used to classify compounds according to their mechanism of action? CIT_{50} data of the ECIS experiment shown in Figure 2 were plotted against compound concentration for each compound. Data were regressed to a linear (blue) and nonlinear (sigmoidal; red) curve. Goodness of fit (R^2) is shown. Mean ± range of one experiment performed in duplicate is shown.

References

1. Delvecchio R, Higa LM, Pezzuto P, Valadão AL, Garcez PP, Monteiro FL, Loiola EC, Dias AA, Silva FJM, Aliota MT, Caine EA, Osorio JE, Bellio M, Connor DHO, Rehen S, Aguiar RS De, Savarino A, Campanati L, Tanuri A. Chloroquine, an endocytosis blocking agent, inhibits Zika virus infection in different cell models. Viruses. 2016;8(322):1–15.

2. Mounce BC, Cesaro T, Carrau L, Vallet T, Vignuzzi M. Curcumin inhibits Zika and chikungunya virus infection by inhibiting cell binding. Antiviral Res. 2017;142:148–57.

3. Tan CW, Sam IC, Chong WL, Lee VS, Chan YF. Polysulfonate suramin inhibits Zika virus infection. Antiviral Res. 2017;143:186–94.

4. Alen MMF, de Burghgraeve T, Kaptein SJF, Balzarini J, Neyts J, Schols D. Broad Antiviral activity of Carbohydrate-binding agents against the four serotypes of dengue virus in monocyte-derived dendritic cells. PLoS One. 2011;6(6).

5. Rausch K, Hackett BA, Weinbren NL, Reeder SM, Sadovsky Y, Hunter CA, Schultz DC, Coyne CB, Cherry S. Screening bioactives reveals Nanchangmycin as a broad spectrum antiviral active against Zika virus. Cell Rep. 2017;18(3):804–15.

6. Varghese FS, Rausalu K, Hakanen M, Saul S, Kümmerer BM, Susi P, Merits A, Ahola T. Obatoclax inhibits alphavirus membrane fusion by neutralizing the acidic environment of endocytic compartments. Antimicrob Agents Chemother. 2016;61(3):1–17.

7. Oeyen M, Sam N, Sandra C, Myrvold BO, Schols D. A unique class of lignin derivatives displays broad anti-HIV activity by interacting with the viral envelope. Virus Res. 2019;274.

8. Konkolova E, Dejmek M, Hrebabecky H, Sala M, Böserle J, Nencka R, Boura E. Remdesivir triphosphate can efficiently inhibit the RNA-dependent RNA polymerase from various flaviviruses. Antiviral Res. 2020;182:10–3.

9. Kamiyama N, Soma R, Hidano S, Watanabe K, Umekita H, Fukuda C, Noguchi K, Gendo Y, Ozaki T, Sonoda A, Sachi N, Runtuwene LR, Miura Y, Matsubara E, Tajima S, Takasaki T, Eshita Y, Kobayashi T. Ribavirin inhibits Zika virus (ZIKV) replication in vitro and suppresses viremia in ZIKV-infected STAT1-deficient mice. Antiviral Res. 2017;146:1–11.

10. Sacramento CQ, Melo GR De, Freitas CS De, Marttorelli A, Ferreira AC, Barbosa-lima G, Volotão M, Nunes EP, Tschoeke DA, Leomil L, Loiola EC, Trindade P, Rehen SK, Bozza FA, Bozza PT, Boechat N, Thompson FL, de Filippis AMB, Brüning K et al. The clinically approved antiviral drug sofosbuvir inhibits Zika virus replication. Sci Rep. 2017;1–12.
